# Unveiling the Cellular Mystery of Isoniazid–Rifampicin (IR) in CYP2E1 Overexpressing HepG2 Cells

**DOI:** 10.1155/ijh/9275461

**Published:** 2026-11-03

**Authors:** Debasree Bishnu, Suman Santra, Partha Sarathi Mukherjee, Gopal Krishna Dhali, Abhijit Chowdhury, Amal Santra

**Affiliations:** ^1^ Department of Basic Science and Disease Biology John C. Martin Center for Liver Research and Innovations Kolkata India; ^2^ Department of Hepatology School of Digestive and Liver Diseases School of Digestive and Liver Diseases Institute of Post Graduate Medical Education & Research Kolkata India ipgmer.gov.in; ^3^ Department of Gastroenterology School of Digestive and Liver Diseases Institute of Post Graduate Medical Education & Research Kolkata India ipgmer.gov.in

**Keywords:** antitubercular drugs, apoptosis, drug-induced liver injury, lysosomal destabilization, mitochondrial membrane potential (MMP), NOX

## Abstract

**Background and Aims:**

The molecular basis of liver injury observed following cotreatment with the first‐line antitubercular drugs (ATDs) isoniazid (INH) and rifampicin (RIF) is not fully understood. Cytochrome P450 2E1 (CYP2E1), a key enzyme in drug metabolism, is thought to contribute to INH‐RIF (IR)–associated hepatotoxicity. In this study, we investigated the intracellular events, including lysosomal and mitochondrial alterations, that occur in CYP2E1 overexpressing HepG2 (HepG2‐E47) cells during IR exposure.

**Methods:**

HepG2‐E47 cells overexpressing CYP2E1 were cotreated with 5 *μ*M INH and 2.5 *μ*M RIF for various time points. Reactive oxygen species (ROS) generation, antioxidant defense status, and CYP2E1 activity were assessed. Lysosomal destabilization, mitochondrial depolarization, and cell death were analyzed using fluorescence spectrophotometer and flow cytometry. To explore CYP2E1 involvement, cells were pretreated with the selective CYP2E1 inhibitor chlormethiazole (CMZ). Pharmacological modulators were used to explore the role of lysosomal and mitochondrial involvement following IR treatment.

**Results:**

IR treatment significantly enhanced CYP2E1 activity, ROS production, and impairment of the cellular antioxidant system. These effects were prevented by CMZ, indicating CYP2E1‐dependent oxidative stress. Increased ROS in hepatocytes was accompanied by an early lysosomal dysfunction and cathepsin D release, which were followed by mitochondrial depolarization and apoptosis of the cells. Treatment with bafilomycin, a pharmacological modulator of lysosomal function, alleviated mitochondrial damage, consistent with lysosomal destabilization preceding mitochondrial dysfunction.

**Conclusion:**

CYP2E1 plays a pivotal role in IR‐associated cellular injury in CYP2E1 overexpressing HepG2 cells by driving oxidative stress that triggers lysosomal and mitochondrial dysfunction leading to apoptosis. Targeting CYP2E1 or its downstream signalling pathways may provide therapeutic potential against ATD‐associated liver injury.

## 1. Introduction

Drug‐induced liver injury (DILI) remains a major global health issue and is one of the leading causes of liver‐related morbidity and mortality [1, 2]. Among the agents responsible, antitubercular drugs (ATDs) are a predominant cause, contributing to approximately 46%–58% of DILI cases in India, the highest incidence reported worldwide [3]. This variation across regions may be attributed to differences in tuberculosis prevalence, treatment regimens, genetic factors, and pharmacovigilance practices. The particularly high burden of ATD‐associated hepatotoxicity in India highlights the urgent need to elucidate the underlying molecular mechanisms.

Among first line ATDs, isoniazid (INH) and rifampicin (RIF) are the primary drugs implicated in liver injury [4]. Although their combined use enhances the therapeutic efficacy of tuberculosis treatment and prevents drug resistance, it substantially increases the risk of hepatotoxicity. Clinically, INH is the most potent hepatotoxin among ATDs, capable of causing hepatocellular damage ranging from asymptomatic elevation of serum transaminases to acute liver failure with high mortality [5–7]. RIF, commonly used alongside INH in tuberculosis treatment, enhances INH‐related liver damage [4, 8–12]. The liver, being the major organ for xenobiotic metabolism, is particularly susceptible to INH‐induced injury. Hepatocytes, which are rich in drug metabolizing enzymes, play a central role in the biotransformation of INH. During this process, INH is acetylated by N‐acetyltransferase 2 (NAT2) to form acetyl INH, which is subsequently hydrolyzed to acetyl hydrazine. Acetyl hydrazine is then oxidized by cytochrome P450 2E1 (CYP2E1), leading to the formation of reactive metabolites and free radicals capable of damaging cellular macromolecules [13–15].

Overexpression of CYP2E1 in cells may enhance oxidative stress by promoting the accumulation of reactive oxygen species (ROS), which in turn deplete intracellular glutathione (GSH), disrupt antioxidant defenses, and initiate lipid peroxidation [16, 17]. Both clinical and experimental findings consistently support oxidative stress as a central mechanism in ATD‐related hepatotoxicity [18, 19]. As CYP2E1 is highly abundant in hepatocytes, this enzyme plays a dual role: vital for drug metabolism under normal conditions but deleterious when overactivated. RIF further aggravates this effect by inducing CYP2E1 expression, thereby intensifying ROS generation and amplifying INH‐mediated hepatocellular injury.

In liver injury, organelle dysfunction, particularly of lysosomes and mitochondria, contributes significantly to hepatocyte damage. Lysosomal perturbation releases cathepsins that act as proapoptotic mediators, amplifying oxidative stress and triggering downstream mitochondrial impairment [20–22]. Mitochondria, central regulators of energy metabolism and apoptosis, undergo depolarization in response to ROS, further promoting hepatocyte death [23–25]. Despite these insights, the precise sequence of events connecting CYP2E1‐mediated ROS production, lysosomal and mitochondrial dysfunction, and hepatocyte death during ATD‐associated liver injury remains poorly understood. There is no validated pharmacological strategy currently exists to prevent ATD‐associated hepatotoxicity without compromising antitubercular efficacy. Identifying the specific intracellular sequence of injury and in particular, whether lysosomal or mitochondrial dysfunction represents the earlier, more therapeutically tractable target has direct relevance for developing adjunct hepatoprotective strategies for patients on standard first‐line TB regimens.

In this study, we used the HepG2‐E47 cell line, which stably overexpresses human CYP2E1, because standard HepG2 cells express hepatic CYP enzymes at low and variable levels, limiting their utility for isolating CYP‐dependent bioactivation mechanisms. Stable CYP2E1 overexpression in this genetic background allows CYP2E1 specific contributions to oxidative injury to be examined without the confounding enzyme variability of native HepG2.

We hypothesized that CYP2E1‐generated ROS is the initiating event in IR‐associated hepatocellular injury, and that this oxidative insult triggers lysosomal destabilization as an early, upstream event that subsequently drives mitochondrial depolarization and apoptotic cell death—rather than lysosomal and mitochondrial dysfunction occurring in parallel or in the reverse order. We tested this hypothesis using pharmacological inhibition of CYP2E1 (chlormethiazole [CMZ]) and modulators of lysosomal (bafilomycin A1 [BafA] and chloroquine [CHQ]) and mitochondrial (cyclosporin A [CsA] and diazoxide [DIA]) function in CYP2E1 overexpressing HepG2 cells.

## 2. Materials and Methods

### 2.1. Cell Line and Culture

The HepG2‐E47 cell line (RRID: CVCL_A2HG) was kindly provided as a gift by Professor Arthur I. Cederbaum of Icahn School of Medicine at Mount Sinai, New York. This human hepatoma cell line, stably expressing human CYP2E1 was used in this study to investigate the intracellular mechanisms of INH‐RIF (IR)–induced hepatotoxicity. As CYP2E1 is a major hepatic Phase I drug metabolizing enzyme responsible for the bioactivation of INH and generation of ROS, this model is particularly suited to study CYP2E1‐mediated oxidative stress and DILI. HepG2‐E47 cells were maintained in minimum essential medium (MEM) supplemented with 10% fetal bovine serum (FBS), 0.5 mg/mL G418, 100‐U/mL penicillin, and 100‐*μ*g/mL streptomycin. Cells were cultured at 37°C in a humidified incubator with 5% CO_2_. MEM and antibiotics used for culture were obtained from Sigma‐Aldrich (United States) and FBS was procured from Gibco, Thermo Fisher, United States.

### 2.2. Treatment Regimen of INH and RIF for HepG2‐E47 Cells and Use of Pharmacological Interventions

At 70% confluency, HepG2‐E47 cells (1 × 10^5^ cells/mL) were cotreated with 5 *μ*M INH and 2.5 *μ*M RIF (IR) for different time periods (1–24 h) to assess intracellular ROS generation, cellular antioxidant status, cellular CYP2E1 activity, lysosomal function, mitochondrial integrity study, and mode of cell death. In some separate experiments, we used different pharmacological modulators to understand the involvement of different cellular organelles in the initiation of cellular injury related to IR treatment. To examine the contribution of CYP2E1, a subset of cells was preincubated with the selective CYP2E1 inhibitor CMZ (50 *μ*M) for 3 h prior to IR administration. To investigate the involvement of lysosomal function in IR‐induced cellular injury, cells were pre‐exposed to BafA (10 *μ*M) or ChQ (20 *μ*M), pharmacological modulators of lysosomal stability, for 30 min before IR treatment. In separate experiments investigating mitochondrial involvement, cells were pretreated with CsA (10 *μ*M) and DIA (50 *μ*M), agents known to preserve mitochondrial integrity, prior to IR administration. All pharmacological agents used in these interventions were obtained from Sigma‐Aldrich (United States). CMZ, BafA, CsA, and DIA were dissolved in dimethyl sulfoxide (DMSO), whereas ChQ was dissolved directly in culture medium. Corresponding vehicle controls were included for the respective treatments, with DMSO‐treated cells serving as vehicle controls for CMZ, BafA, CsA, and DIA, and culture medium‐treated cells serving as the control for ChQ. The final concentration of DMSO kept in the culture medium was less than 0.1%. Sample sizes as shown in the legends reflect the number of independent replicate experiments performed for each assay. Assay requiring flow cytometric acquisition were limited (*n* = 3) by instrument availability, whereas colorimetric/kit–based assays (*n* = 5) were performed. However, in each experiment triplicate samples were used.

### 2.3. CYP2E1 Activity

To assess CYP2E1‐dependent bioactivation of IR, this enzymatic activity was evaluated as described in our previous work [26] using the p‐nitrophenol (PNP) hydroxylation assay [27]. Enzyme activity was expressed as the rate of PNP hydroxylation per milligram of protein.

### 2.4. Detection of Intracellular ROS by 2 ^′^,7 ^′^‐Dichlorofluorescin Diacetate (DCFH‐DA) Staining

ROS are unstable bioproducts of cellular metabolism, and their overproduction leads to cellular oxidative stress. Intracellular ROS levels in HepG2‐E47 cells were evaluated using the fluorescent probe DCFH‐DA at different time points [28] by two complementary methods: quantification using fluorescence spectrophotometer and evaluation by flow cytometer using DCF fluorescence as we described previously [26, 29]. We quantitated intracellular ROS by measuring DCF fluorescence intensity in cell lysates (prepared by 0.1% TritonX‐100) using fluorescence spectrophotometer (PerkinElmer) at excitation and emission wavelengths of 488 and 535 nm, respectively, and expressed as arbitrary fluorescence units (AFU). Flow cytometry for detection of intracellular ROS was performed as described previously [26, 29]. Gating strategy, data acquisition, and analysis in flow cytometry were described in Supporting Information file and in Figure S1.

### 2.5. Reduced GSH Measurement

Reduced GSH, a cellular antioxidant that neutralized free radicals and protected the cells from oxidative stress, levels were quantified using the GSH Assay Kit (Catalog # MAK364, Sigma‐Aldrich; United States) performed in kinetic mode at 450 nm following the manufacturer′s instructions.

### 2.6. Lipid Peroxidation Assay

Lipid peroxidation, for assessment of cellular oxidative stress and oxidative damage, was quantified using the MDA Assay Kit (Catalog # MAK085; Sigma‐Aldrich, United States) following the manufacturer′s instructions. MDA levels were calculated from a standard curve and expressed as nanomoles per milligram of protein.

### 2.7. Protein Extraction and Quantification

Whole cell lysates were prepared using RIPA buffer (Cell Signaling Technology, United States) supplemented with protease inhibitor cocktail (Roche Diagnostics, Germany). Protein concentration was determined using the Bradford assay [30] using commercially available Sigma‐Aldrich Kit, United States, according to the manufacturer’s instructions.

### 2.8. Catalase Activity

Catalase is an important antioxidant enzyme responsible for breaking down hydrogen peroxide into water and oxygen molecules. This enzyme activity was assessed spectrophotometrically following the Beers and Sizer method, in accordance with reported protocol [26, 31]. Catalase activity was calculated based on the rate of H_2_O_2_ decomposition.

### 2.9. Superoxide Dismutase (SOD) Activity

SOD is an antioxidant enzyme responsible for protecting the cells from superoxide radicals into hydrogen peroxide and molecular oxygen. This enzyme activity was measured according to the method of Nandi and Chatterjee [32], based on the ability of SOD to inhibit pyrogallol autoxidation [26]. Specific activity was expressed as units per minute per milligram protein.

### 2.10. NADPH Oxidase (NOX) Activity

NOX is a family of membrane‐bound enzymes that generate ROS in the cells. This enzyme activity was measured as described previously [26, 33]. NOX activity was calculated using the extinction coefficient for NADPH (6.22 mM^−1^ cm^−1^).

### 2.11. Lysosomal Stability Assay

Lysosomal stability assay was used to test whether the integrity of lysosomes was compromised before mitochondrial depolarization as we predicted in our hypothesis. This assay was carried out using LysoTracker Red (LTR), a fluorescent dye that selectively accumulates in acidic lysosomes [34]. Cells were incubated with 50 nM (LTR) (Cat # L7528; Molecular Probes, United States) for 15 min at 37°C under dark conditions to prevent photobleaching. After staining, cells were washed with PBS, harvested, and resuspended in PBS for analysis. Fluorescence intensity of LTR was measured using a BD FACSCalibur (BD Biosciences, Pharmingen, United States) flow cytometer with detection in the FL3 channel. LTR fluorescence was also quantified using a fluorescence spectrophotometer with excitation and emission wavelengths set at 577 and 590 nm, respectively.

### 2.12. Cathepsin Activity Assay

To further evaluate lysosomal function, cathepsin B and cathepsin D activities were quantified using fluorometric enzyme activity kits (cathepsin B: BioVision K140‐100; cathepsin D: BioVision K143‐100) as per instruction of the manufacturer. Enzyme activity was expressed as relative fluorescence units per microgram of protein.

### 2.13. Mitochondrial Dysfunction Assay

Mitochondrial dysfunction was assessed by studying mitochondrial membrane potential (MMP) using the cationic dye Rhodamine 123 (RH‐123) [35] with slight modification. To assess MMP, briefly cells were incubated with 16.6 *μ*M RH‐123 for 30 min at 37°C before completion of treatment. After staining, cells were washed twice with PBS to remove excess dye, trypsinized, centrifuged, and resuspended in PBS for flow cytometric analysis (BD FACSCalibur; BD Biosciences, Pharmingen, United States). Fluorescence intensity was recorded in the FL1 channel, and 10,000 gated cells were analyzed per sample. Mitochondrial depolarization was interpreted as a shift toward lower fluorescence intensity relative to control cells.

### 2.14. Annexin V–Propidium Iodide (PI) Staining and Flow Cytometric Analysis of Apoptosis

Apoptotic cell death following IR exposure was assessed using Annexin V and PI staining [29]. After the designated treatment period, cells were harvested, washed twice with ice‐cold PBS, and resuspended in binding buffer at a concentration of 1 × 10^5^ cells/mL. Staining was performed using the Annexin V–Fluos kit (Roche, Germany) according to the manufacturer′s instructions. Fluorescence was recorded for 10,000 cells using a BD FACSCalibur (BD Biosciences, Pharmingen, United States) flow cytometer, with annexin V detected in the FL1 channel and PI detected in the FL2 channel, allowing discrimination of apoptotic and necrotic populations.

### 2.15. Caspase 3 Activity Assay

Caspases are a family of protease enzymes responsible for execution programme cell death. Caspase 3 activity in HepG2‐E47cells was measured using a fluorometric assay kit (Catalog # K105‐100; BioVision, United States) according to the manufacturer′s instructions as mentioned earlier [29].

### 2.16. Statistical Analysis

Data are expressed as mean ± standard deviation. Comparisons between two groups were conducted using an unpaired two‐tailed Student′s *t*‐test, whereas analyses involving more than two groups were performed using ANOVA. For all experiments, *p* < 0.05 was considered statistically significant. All statistical analyses were carried out using GraphPad Prism 8 software (GraphPad, United States).

## 3. Results

### 3.1. IR Cotreatment Is Associated With Increased CYP2E1 Activity and Oxidative Stress in HepG2‐E47 Cells

Cellular CYP2E1 activity remained at a steady state in HepG2‐E47 cells without IR treatment, whereas IR treatment caused a progressive increase of cellular CYP2E1 activity (Figure 1A). This increase of CYP2E1 activity during IR treatment was found to be time dependent. Increased cellular CYP2E1 level is known to generate increase in ROS, a mechanism thought to be implicated in ATD‐induced hepatotoxicity. We therefore assessed intracellular ROS in HepG2‐E47 cells during IR treatment. Spectrofluorometric analysis of DCF fluorescence in IR‐treated cells revealed higher fluorescence intensity compared with untreated cells (Figure 1B). Increase of DCF fluorescence in the cells is an indicator of intracellular ROS generation. During the first 3 h of IR treatment, intracellular ROS was sharply increased, whereas from 6 to 24 h, the increased ROS remained relatively steady (Figure 1B). Flow cytometric analysis further revealed a time‐dependent increase of intracellular ROS during IR treatment compared with control cells (Figure 1C). In the representative histograms of flow cytometry, we noted two populations of cells, M1 (low ROS‐generating cells) and M2 (high ROS‐generating cells). With advanced periods of IR treatment, there was increased M2 population of cells, indicating increased number of cells producing ROS (Figure 1C). Machine‐generated data obtained from flow cytometry for control and IR treatment is depicted in Figure S2 after acquisition of 10,000 cells for each experiment. These results indicate that IR treatment is associated with CYP2E1 activation, leading to excessive ROS generation and oxidative stress in HepG2‐E47 cells.

**Figure 1:**
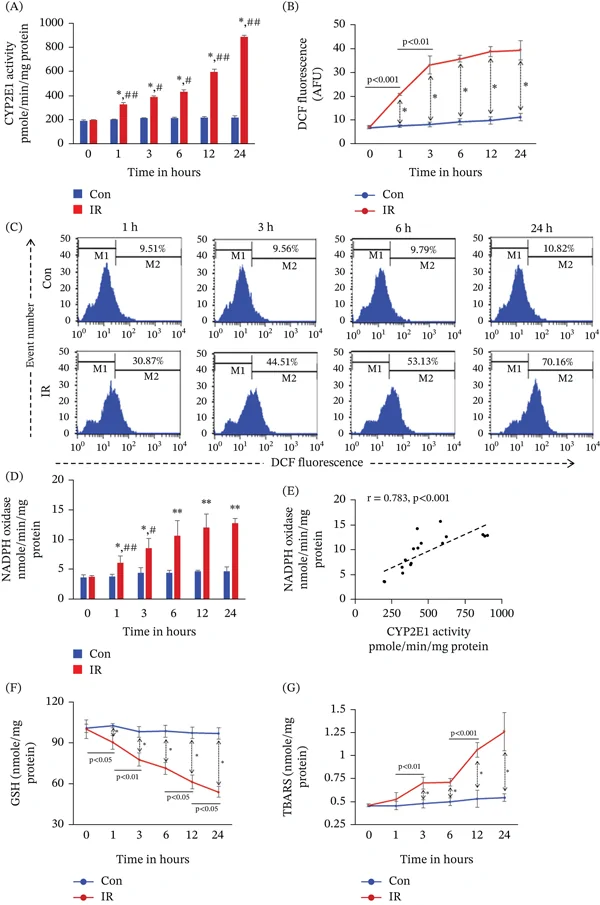
INH‐RIF cotreatment was associated with CYP2E1 activation and increased oxidative stress in HepG2‐E47 cells. (A) CYP2E1 activity was measured in HepG2‐E47 cells with or without IR treatment for the indicated time periods. IR cotreatment resulted in a progressive increase in CYP2E1 activity compared with untreated controls (*n* = 3).  ^∗^ *p* < 0.001 compared with respective control cells; #*p* < 0.01 compared with previous time points in IR‐treated cells; ##*p* < 0.001 compared with previous time points in IR‐treated cells. (B) Intracellular ROS generation was assessed by spectrofluorometric measurement of DCF fluorescence derived from DCFH‐DA, which was expressed in arbitrary fluorescence units (AFU). A pronounced increase in DCF fluorescence was observed during the first 3 h of IR treatment, followed by a plateau phase from 6 to 24 h, indicating sustained ROS levels over the treatment period (*n* = 3).  ^∗^ *p* < 0.001 compared with respective control cells. (C) ROS levels were further assessed by flow cytometry using the fluorescent probe DCFH‐DA. Representative flow cytometric histograms show DCF fluorescence intensity at different time points. A progressive shift in fluorescence from M1 (low ROS producing cells) to M2 (high ROS producing cells) indicates an increase in ROS positive cell populations following IR treatment. (D) NADPH oxidase activity increased progressively in IR‐treated cells, suggesting enhanced ROS formation associated with CYP2E1 induction (*n* = 5).  ^∗^ *p* < 0.01 compared with respective control cells;  ^∗∗^ *p* < 0.001 compared with respective control cells; #*p* < 0.05 compared with previous time point in IR‐treated cells; ##*p* < 0.01 compared with previous time point in IR‐treated cells. (E) Correlation between increased CYP2E1 activity and NADPH oxidase activity in HepG2‐E47 cells during IR treatment, showed a positive correlation (*r* = 0.783; *p* < 0.001) indicating CYP2E1‐dependent ROS generation (*n* = 3). (F) Intracellular‐reduced glutathione (GSH) levels declined significantly in IR‐treated cells in a time‐dependent manner, whereas untreated control cells maintained stable levels (*n* = 5).  ^∗^ *p* < 0.001 compared with respective control cells. (G) Lipid peroxidation, determined by thiobarbituric acid reactive substances (TBARS) levels, increased markedly after IR exposure, indicating development of oxidative stress within the cell (*n* = 5).  ^∗^ *p* < 0.001 compared with respective control cells. Data represent mean ± SD.

Among the different sources of ROS in hepatocytes, NOXs are one of the important sources [36–38]. We therefore assessed NOX activity in HepG2‐E47 cells during IR treatment and found it to be progressively increased with duration of IR exposure (Figure 1D). We therefore tried to correlate NOX and CYP2E1 activities in HepG2‐E47 cells during IR treatment. We observed a positive correlation (*r* = 0.783; *p* < 0.001) between NOX and CYP2E1 activities during IR treatment (Figure 1E).

### 3.2. CYP2E1‐Mediated Redox Imbalance Following IR Cotreatment in HepG2‐E47 Cells

Intracellular GSH level, a key indicator of cellular redox status [39], plays an important role in cellular defense system. To evaluate the impact of IR‐induced ROS on the cellular antioxidant system, key redox biomarkers were assessed. The antioxidant network comprises enzymatic and nonenzymatic components that work collectively to maintain redox homeostasis. Among nonenzymatic defenses, GSH serves as a major redox buffer, whereas lipid peroxidation products, TBARS, reflect oxidative damage to membrane lipids [40]. In HepG2‐E47 cells, GSH level remained stable for 24 h without IR treatment; in contrast cells with IR treatment showed a progressive decline of GSH content (Figure 1F). Parallel to the downfall of cellular GSH content, TBARS level was found to be elevated in IR‐treated cells (Figure 1G) indicating development of cellular oxidative stress due to IR treatment. These findings provoked us to study antioxidant enzymes particularly SOD and catalase during treatment of the cells. Among the enzymatic ROS scavengers, SOD serves as the first line of defense by rapidly dismutating superoxide radicals [41]. SOD activity in HepG2‐E47 cells during IR treatment fell progressively from 125.64 ± 3.78 U at 0 h to 54.09 ± 1.98 U at 24 h (Table 1). But SOD activity of control cells remained unchanged during this time period (Table 1). Cellular catalase has a high turnover rate and acts as a crucial secondary defense by decomposing hydrogen peroxide [42]. During first 6 h of IR treatment in HepG2‐E47 cells, catalase levels progressively increased followed by sharp decline with further progression of treatment (Table 1).

**Table 1:** Time‐Dependent Changes in SOD and Catalase Activities in HepG2‐E47 Cells Following IR Co‐treatment.

Hours	SOD Untreated	SOD Treated	Catalase Untreated	Catalase Treated
0 h	120.52 ± 4.45	125.64 ± 3.78	6.77 ± 0.57	7.13 ± 0.52
1 h	118.72 ± 4.00	117.85 ± 3.79	6.85 ± 0.62	7.27 ± 1.10
3 h	115.07 ± 4.23	97.00 ± 5.13 ^∗∗∗^, ###	6.89 ± 0.59	9.42 ± 1.73 ^∗^, #
6 h	115.00 ± 2.10	71.92 ± 2.45 ^∗∗∗^, ###	7.29 ± 0.23	13.10 ± 1.58 ^∗∗∗^, ##
12 h	113.88 ± 2.64	58.91 ± 5.97 ^∗∗∗^, ##	7.52 ± 0.31	7.29 ± 0.37 ###
24 h	113.71 ± 4.14	54.09 ± 1.98 ^∗∗∗^	7.51 ± 0.84	7.42 ± 0.82

Data represent mean ± SD.  ^∗∗∗^p<0.001 vs. untreated;  ^∗^p<0.05 vs. untreated; #p<0.05 vs. treated previous time point; ###p<0.001 vs treated previous time point; ##p<0.01 vs. treated previous time point. n=5.

### 3.3. Inhibition of CYP2E1 Attenuates Oxidative Stress Following IR‐Induced Oxidative Stress Treatment in HepG2‐E47 Cells

Recent review on ATD‐associated hepatotoxicity emerged CYP2E1 as a key enzyme in ATD‐induced hepatotoxicity [43], we therefore pretreated HepG2‐E47 cells with CMZ, a selective CYP2E1 inhibitor [44] to adjudicate the role of CYP2E1 in IR‐associated oxidative stress. CMZ pretreatment effectively suppressed IR‐related elevation of CYP2E1 levels in HepG2‐E47 cells (Figure 2A); significantly reduced intracellular ROS as evident by flowcytometry histograms of DCF fluorescence that showed shifting of higher fluorescence signals due to IR treatment to lower fluorescence signals like control cells (Figure 2B). The original histograms along with the data originated from flow cytometer are depicted in Figure S3. This finding indicated that upon IR treatment, increase in ROS is strongly dependent on CYP2E1 activity.

**Figure 2:**
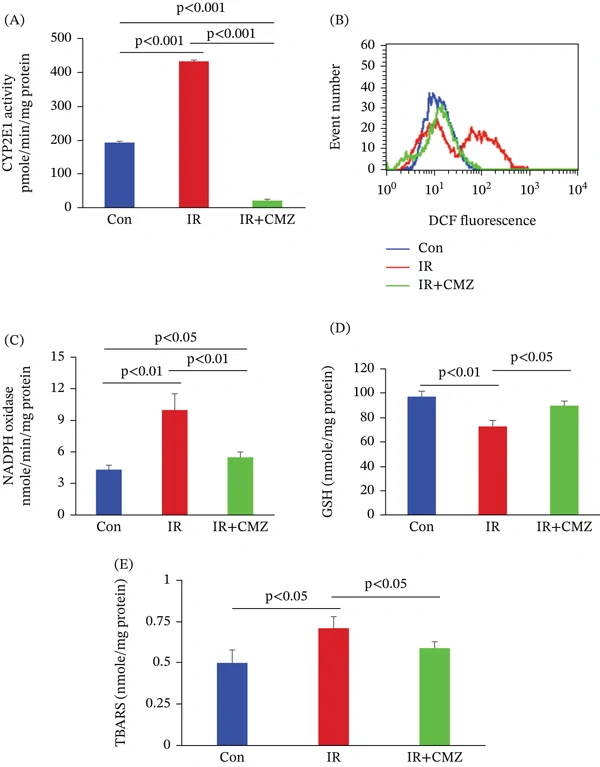
Inhibition of CYP2E1 by CMZ attenuated INH‐RIF–associated oxidative stress in HepG2‐E47 cells. All experiments were performed after 6 h of IR treatment. (A) CYP2E1 activity measured in untreated control, IR, and CMZ pretreated (IR + CMZ) cells. Pretreatment with CMZ significantly suppressed the IR‐mediated increase in CYP2E1 activity (*n* = 3). (B) Representative flow cytometric histogram showing overlapping DCF fluorescence profiles of untreated control, IR‐treated, and IR + CMZ–treated HepG2‐E47 cells. ROS generation, indicated by a rightward shift in DCF fluorescence following IR treatment, was attenuated by CMZ pretreatment (*n* = 3). (C) NADPH oxidase activity, elevated by IR treatment, was reduced by CMZ pretreatment (*n* = 3). (D) Depletion of intracellular‐reduced glutathione (GSH) by IR was restored to near control levels with CMZ (*n* = 3). (E) Lipid peroxidation, measured as thiobarbituric acid reactive substances (TBARS), was reduced in CMZ pretreated cells compared with IR‐treated cells (*n* = 3). Data represent mean ± SD.

We next assessed cellular NOX activity, GSH levels, and lipid peroxidation in HepG2‐E47 cells which were pretreated with CMZ before IR treatment. Notably, CMZ pretreatment effectively reversed these changes, reducing NOX activity (Figure 2C), restoring GSH content (Figure 2D) and TBARS level reverting back to near control values (Figure 2E). All these findings suggest that CYP2E1 activation is a critical event driving IR‐associated oxidative stress in HepG2‐E47 cells and pharmacological inhibition of CYP2E1 with CMZ mitigates ROS generation, preserves cellular redox homeostasis, and protects against lipid peroxidation, underscoring the central role of CYP2E1 in mediating the hepatotoxic effects of IR cotreatment.

### 3.4. IR Results in Early Lysosomal Damage

Having established that IR treatment activates CYP2E1‐dependent ROS production, we next examined whether this oxidative stress selectively and preferentially destabilizes lysosomes ahead of mitochondrial injury as predicted by our hypothesis. In hepatocytes, lysosomes play a pivotal role in cellular defense and injury process. Toxic metabolites of xenobiotics, increased intracellular ROS generation, accumulation of lipid, alcohol intake and toxins may disrupt lysosomal integrity leading to release of enzymes that ultimately cause cell death [45, 46]. To evaluate the effect of IR treatment on lysosomal stability, we used the lysosomotropic fluorescent probe LTR and performed a time‐dependent flow cytometric analysis in HepG2‐E47 cells. Figure 3A depicts the representative histograms of LTR fluorescence of HepG2‐E47 cells treated with or without IR in different time points. In these histograms, according to the intensity of fluorescence, cells are distributed into 2 zones. M1 zone in which cells had low fluorescence indicating pale cells with lysosomal damage. Cells in M2 zone had high LTR fluorescence and the cells were healthy with intact lysosomes. Untreated or control cells had stable LTR high fluorescence throughout the experiment and 97.46*%* ± 2.76*%* cells were healthy at 90 min. But when the cells were treated with IR, pale cell population was increased from 45 min and became markedly pronounced by 90 min (Figure 3A). Cells having intact lysosomes were reduced to 69.56*%* ± 4.12*%*, *p* < 0.001 at 90 min in IR‐treated group. The machine‐generated original histograms and data are shown in Figure S4. Pretreatment with CMZ, an inhibitor of CYP2E1, significantly reduced IR‐mediated M1 cells population (Figure 3B), suggesting effective protection of lysosomal damage for IR treatment. Original histograms and data obtained in flow cytometer are shown in Figure S5.

**Figure 3:**
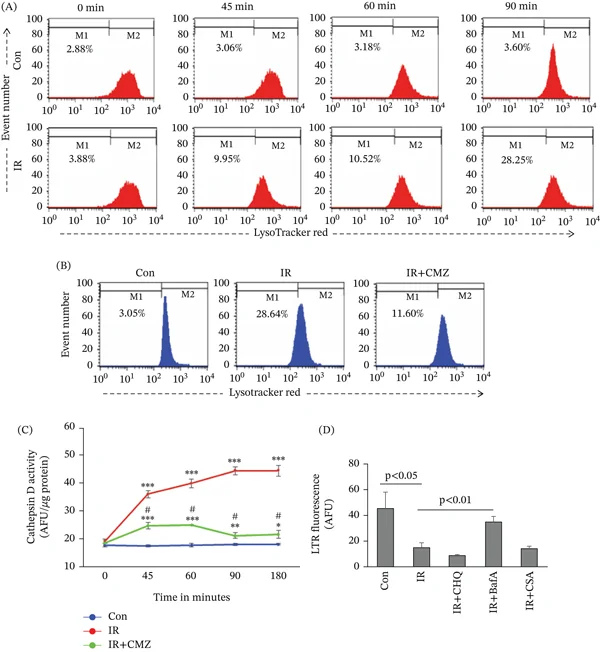
INH‐RIF cotreatment promoted early lysosomal dysfunction in HepG2‐E47 cells through a CYP2E1‐dependent mechanism. (A) Representative flow cytometric histograms showing LTR fluorescence over time. IR treatment resulted in a progressive reduction in LTR intensity, indicating lysosomal destabilization. Cells in the M1 region represent low fluorescence (damaged lysosomes or pale cell) populations, whereas cells in the M2 region represent high fluorescence (healthy lysosomes) populations. (B–D) Experiments were performed following 90 min of IR treatment (*n* = 3). (B) Representative histograms depicting LTR fluorescence in untreated control, IR‐treated, and IR + CMZ–treated HepG2‐E47 cells. Pretreatment with the CYP2E1 inhibitor chlormethiazole (CMZ) markedly prevented the IR‐associated loss of LTR fluorescence, suggesting that CYP2E1 activation contributes to lysosomal damage. (C) Cathepsin D activity, an indicator of lysosomal injury, increased in a time‐dependent manner following IR treatment, and this was suppressed by CMZ pretreatment.  ^∗^ *p* < 0.05 compared with respective control cell;  ^∗∗^ *p* < 0.01 compared with respective control cell;  ^∗∗∗^ *p* < 0.001 compared with respective control cells; #*p* < 0.001 compared with respective IR‐treated cells. (D) Quantification of lysosomal integrity by LTR fluorescence after IR treatment with or without pharmacological modulators. IR reduced LTR intensity, indicating lysosomal destabilization. Chloroquine (CHQ) (lysosomotropic agent) further enhanced LTR loss, whereas bafilomycin A1 (BafA) (pharmacological modulator) preserved LTR fluorescence. Cyclosporin A (CsA) (mitochondrial stabilizer) showed no protective effect. Data represent mean ± SD.

To evaluate lysosomal involvement, cathepsin B and D activities were measured to evaluate cathepsin release. As shown in Figure 3C, IR treatment produced a significant increase in cathepsin D activity over the course of the experiment, whereas CMZ pretreatment substantially prevented this elevation. These findings indicate that lysosomal destabilization occurs early and is closely linked to CYP2E1‐dependent oxidative stress. In contrast IR treatment did not produce any noticeable change in cathepsin B activity **(**23.81 ± 2.3 − A.F.U/*μ*g protein at 0 min to 27.37 ± 2.41 − A.F.U/*μ*g protein at 180 min; *p* > 0.05) across the time course of the experiment.

To delineate the sequence of organelle dysfunction following IR treatment, pharmacological modulators targeting lysosomal and mitochondrial pathways were employed, and lysosomal integrity was quantified by measuring LTR fluorescence intensity (Figure 3D). Control cells exhibited high LTR fluorescence, indicating intact lysosomes. Exposure to IR markedly reduced LTR fluorescence, reflecting lysosomal destabilization. This reduction was further enhanced by pretreatment with CHQ, a lysosomotropic agent [47] known to promote lysosomal rupture. In contrast, pretreatment with BafA, a pharmacological modulator [46, 48], restored LTR fluorescence to near control levels, suggesting preservation of lysosomal integrity. CsA, a mitochondrial stabilizer [49], failed to prevent IR‐associated LTR loss, consistent with lysosomal destabilization occurring upstream of mitochondrial dysfunction.

### 3.5. IR Is Linked to Mitochondrial Membrane Depolarization Downstream of Lysosomal Damage in HepG2‐E47 Cells

Mitochondria are vital for liver cell functions, including energy production, redox balance, calcium regulation, and metabolism, while also playing key roles in cell death [50, 51]. Mitochondrial dysfunction is characterized by loss of MMP, opening of the mitochondrial permeability transition (MPT) pore, and release of intermembrane proteins into the cytoplasm [51, 52]. However, the mechanisms underlying mitochondrial dysfunction during IR‐related liver toxicity remain unclear, prompting investigation into mitochondrial membrane depolarization following lysosomal damage in HepG2‐E47 cells. MMP was measured using Rh123, a cationic fluorescent dye that accumulates in active mitochondria. Flow cytometric analysis showed that IR exposure led to a gradual decline in Rh123 fluorescence in HepG2‐E47 cells, indicating progressive mitochondrial depolarization (Figure 4A). Original histograms and data generated from flow cytometer are shown in Figure S6. The proportion of cells with reduced MMP (M1 population) increased markedly from 1 h onward, reaching approximately 60% by 24 h, whereas untreated control cells maintained stable fluorescence. These findings indicate that IR cotreatment is associated with significant mitochondrial dysfunction in CYP2E1‐expressing hepatocytes. To determine whether this mitochondrial depolarization is mediated by CYP2E1‐derived oxidative stress, cells were pretreated with CMZ which markedly reduced the percentage of cells with low MMP following IR exposure (Figure 4B), suggesting the involvement of CYP2E1 in mitochondrial injury. Representative original histograms and data obtained from flow cytometer are depicted in Figure S7.

**Figure 4:**
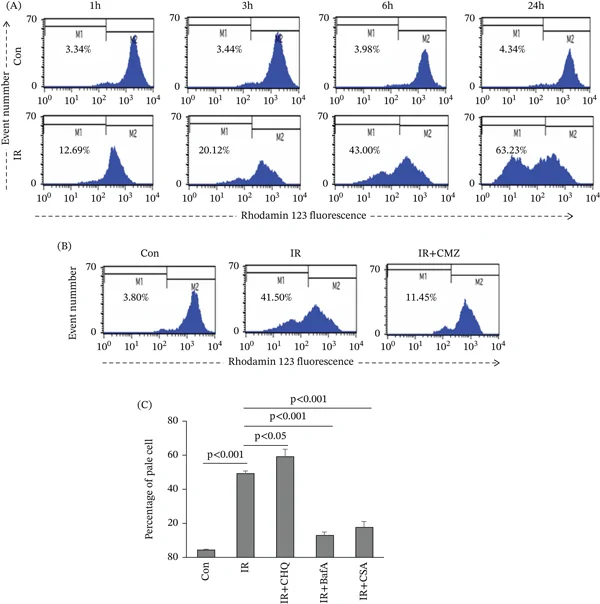
INH‐RIF (IR) cotreatment is accompanied by CYP2E1‐dependent mitochondrial membrane potential (MMP) loss in HepG2‐E47 cells. (A) Representative flow cytometric histograms illustrating changes in MMP in HepG2‐E47 cells following IR treatment for the indicated times (1–24 h). IR exposure resulted in a time‐dependent decrease in Rhodamine 123 fluorescence, indicating mitochondrial depolarization. Control cells maintained stable fluorescence throughout the experiment. M1 represents cells with low Rhodamine 123 fluorescence (mitochondrial depolarization or pale cells), whereas M2 represents cells with high fluorescence intensity, corresponding to healthy cells with intact mitochondria (*n* = 3). (B) Quantitative analysis of the percentage of cells showing MMP loss in untreated, IR‐treated, and IR + CMZ–treated HepG2‐E47 cells. Pretreatment with the CYP2E1 inhibitor chlormethiazole (CMZ) significantly prevented IR‐related MMP dissipation following 6 h, supporting the role of CYP2E1‐mediated oxidative stress in mitochondrial dysfunction (*n* = 3). (C) Quantitative analysis of MMP loss showing that pretreatment with BafA significantly prevented IR‐associated mitochondrial depolarization, whereas CHQ further increased the percentage of cells with low MMP consistent with its lysosomotropic and destabilizing effects. CsA also effectively prevented MMP dissipation, indicating that mitochondrial dysfunction occurs downstream of lysosomal destabilization (*n* = 3). Data represent mean ± SD.

Given the early occurrence of lysosomal destabilization and cathepsin D release in HepG2‐E47 cells, we hypothesized that mitochondrial damage may be a downstream consequence of lysosomal injury. To examine this, cells were pretreated with BafA, ChQ, or CsA prior to IR exposure and MMP was assessed 6 h later using Rh123. As illustrated in the quantitative bar graph, BafA pretreatment significantly prevented MMP loss, as reflected by a reduction in the proportion of cells with low MMP (Figure 4C), whereas ChQ exacerbated it (Figure 4C) implicating that lysosomal destabilization occurs upstream of mitochondrial dysfunction. In contrast, CsA prevented mitochondrial depolarization but did not prevent lysosomal damage, further supporting that lysosomal dysfunction is an upstream event that precipitates secondary mitochondrial dysfunction. Figure S8 shows representative machine‐generated flow cytometric histograms along with the corresponding analytical data obtained from the instrument.

### 3.6. IR Cotreatment Leads to CYP2E1‐Dependent Mitochondria‐Mediated Apoptosis in HepG2‐E47 Cells

To determine the mode of cell death mediated by IR cotreatment, HepG2‐E47 cells were analyzed by flow cytometry using Annexin V and PI dual staining. Annexin V positivity identifies early apoptotic cells, whereas PI uptake reflects loss of membrane integrity associated with late apoptosis or necrosis. As shown in Figure 5A, IR exposure led to a progressive rise in Annexin V positive cells, increasing from 1.57% at 1 h to 25.6% at 24 h. In addition, the Annexin V^+^/PI^+^ population also increased to approximately 8.5% at 24 h, indicating that a subset of cells had advanced to late‐stage apoptosis. In contrast, untreated controls maintained consistently low Annexin V and PI staining. Flow cytometer‐generated original data and dot plots are shown in Figure S9.

**Figure 5:**
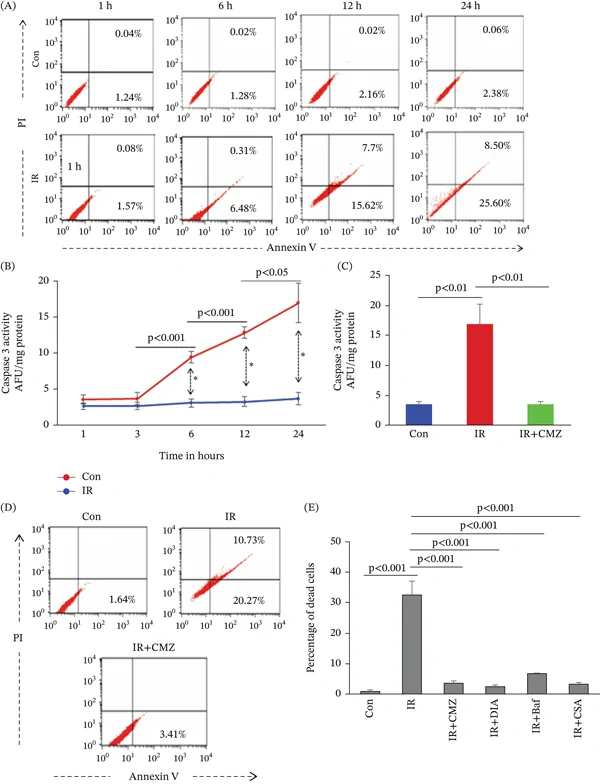
INH‐RIF involves apoptosis through CYP2E1‐mediated mitochondrial dysfunction. (A) Representative flow cytometric dot plots showing annexin V and propidium iodide (PI) dual staining in HepG2‐E47 cells exposed to IR for the indicated time periods. IR treatment led to a time‐dependent increase in annexin V positive cells, indicating progressive apoptotic cell death, whereas untreated control cells displayed minimal staining (*n* = 3). (B) Time‐dependent increase in caspase 3 activity following IR exposure. Caspase 3 activity remained low initially but began to increase significantly from 6‐h post‐IR treatment, reaching a maximum at 24 h, consistent with the kinetics of annexin V positivity (*n* = 5). (C–E) Experiments were performed at 24 h. (C) Effect of CYP2E1 inhibition on IR‐related apoptosis. Pretreatment with CMZ significantly attenuated caspase 3 activation compared with IR alone, suggesting CYP2E1 involvement in IR‐associated apoptosis (*n* = 3). (D) Flow cytometric analysis of annexin V‐FITC/PI–stained cells showing reduced Annexin V positivity upon CMZ pretreatment compared with IR alone, supporting CYP2E1‐dependent apoptotic induction (*n* = 3). (E) Quantification of dead cells in HepG2‐E47 cultures pretreated with BafA, CMZ, CsA or diazoxide (DIA) prior to IR exposure. Pretreatment with BafA, CMZ and CsA significantly reduced IR‐related cell death, indicating that lysosomal stabilization, CYP2E1 inhibition, and mitochondrial protection each mitigate cytotoxicity. DIA pretreatment also conferred protection, further supporting the contribution of mitochondrial dysfunction in IR‐linked hepatotoxicity (*n* = 3). Data represent mean ± SD.

Caspase 3 is an essential executioner enzyme in the apoptotic pathway [53]. To assess apoptosis of HepG2‐E47 cells during IR treatment, caspase 3 activity in whole cell lysate was quantified. Caspase 3 activity showed a gradual increase over time following IR exposure (Figure 5B). It remained low during the early phase but started to rise significantly from 6 h, reaching its peak at 24 h. This pattern aligns with the observed increase in Annexin V positive cells. To determine whether this response was driven by CYP2E1‐mediated oxidative stress, cells were pretreated with the selective CYP2E1 inhibitor CMZ. As shown in Figure 5C, CMZ significantly diminished caspase 3 activation following IR treatment, suggesting the dependence of this apoptotic pathway on CYP2E1 activity. In Figure 5D, flow cytometry analysis evidenced the role of CYP2E1 in apoptosis, as pretreatment with CMZ significantly reduced the number of Annexin V positive cells. Original flow cytometric data and dot plots are presented in Figure S10.

To investigate the contributions of CYP2E1 activity, lysosomal and mitochondrial pathways, and mitochondrial protection in IR‐related cytotoxicity, HepG2‐E47 cells were pretreated with CMZ, BafA, CsA, or DIA before IR (Figure 5E). As graphically represented in the bar plot, pretreatment with CMZ, BafA, and CsA significantly reduced cell death, indicating that inhibition of CYP2E1, lysosomal pathways, and mitochondrial stability provides substantial protection. DIA, another mitochondrial stabilizer [54] also attenuated cell death, highlighting the role of mitochondrial dysfunction. The original flow cytometry data and corresponding dot plots are provided in Figure S11. Collectively, these findings support that IR is linked to apoptosis in HepG2‐E47 cells through a CYP2E1‐dependent mitochondrial pathway, characterized by phosphatidylserine externalization and caspase 3 activation.

## 4. Discussion

Antitubercular DILI remains a major limitation in tuberculosis therapy, with INH and RIF being the principal contributors to hepatotoxicity [55, 56]. Although oxidative stress is widely accepted as a central mechanism underlying IR‐associated liver injury [15, 57], the intracellular events linking drug metabolism to organelle dysfunction and hepatocyte death are not fully defined. Increasing evidence suggests that oxidative stress driven damage to intracellular organelles, particularly lysosomes and mitochondria, plays a critical role in DILI [58, 59], yet the sequence and interdependence of these events during IR exposure remain poorly understood.

This study provides evidence that IR treatment is associated with CYP2E1‐dependent oxidative stress in HepG2 cells, resulting destabilization of lysosomes in an early time period of these drugs treatment. Lysosomal destabilization plays an initiating role in lysosomal‐mitochondrial pathway leading to cell death as evident in this model. To our best knowledge, this is the first detailed attempt to subcellular pathways underlying IR‐related hepatotoxicity in a stable human cell line expressing CYP2E1.

CYP2E1 is uniquely positioned at the intersection of drug metabolism and oxidative injury. The CYP2E1 is a key P450 isoform with a highly uncoupled catalytic cycle that constitutively generates ROS and efficiently bioactivates small hydrazine containing drugs such as INH [60, 61]. Highly expressed in hepatocytes, CYP2E1 is essential for drug metabolism but becomes deleterious when overactivated. Induction of CYP2E1 has therefore been strongly linked to enhanced oxidative stress, lipid peroxidation, and hepatocellular injury [62, 63].

RIF enhances this effect by upregulating CYP2E1, increasing ROS production and exacerbating INH‐related hepatocellular injury [4, 8–12]. Previous clinical and experimental studies have implicated CYP2E1 induction in INH‐related hepatotoxicity [18, 19], and our findings strongly support this association. In this study, cotreatment with INH (5 *μ*M) and RIF (2.5 *μ*M) at doses selected to approximate clinically relevant human serum concentrations produced a marked increase in CYP2E1 activity and ROS generation. The chosen INH concentration (5 *μ*M or 6.8 *μ*g/mL) corresponds to peak serum levels reported in tuberculosis patients [64], whereas the selected RIF concentration represented the minimal dose that produced maximal toxicity when combined with INH, underscoring the translational relevance of the experimental design.

In agreement with these observations, the present study exhibits that IR cotreatment significantly augmented CYP2E1 activity in CYP2E1 overexpressing HepG2 cells, leading to excessive ROS generation. The use of clinically relevant concentrations of INH and RIF strengthens the translational relevance of these findings. Pharmacological inhibition of CYP2E1 using CMZ effectively suppressed ROS accumulation and restored redox balance, supporting that CYP2E1 activation is a primary driver of oxidative stress during IR exposure.

Excessive ROS disrupts cellular redox homeostasis by depleting antioxidant reserves and impairing enzymatic defense systems. GSH, a major intracellular redox buffer, plays a central role in detoxifying reactive intermediates and maintaining cellular homeostasis [65]. Depletion of GSH, increased lipid peroxidation, and altered antioxidant enzyme activity have been widely reported in pathological conditions [65, 66]. Consistent with these reports, IR exposure resulted in progressive depletion of intracellular GSH, elevated lipid peroxidation, and dysregulation of antioxidant enzymes such as SOD and catalase. The transient early increase in catalase activity observed in this study likely reflects an adaptive response to rising hydrogen peroxide levels, which becomes insufficient during sustained oxidative stress. Additionally, the observed increase in NOX activity and its strong positive correlation with CYP2E1 activity suggest that multiple ROS‐generating systems contribute to the overall oxidative burden in hepatocytes during IR exposure, as previously reported in liver injury models [67, 68].

Lysosomes have emerged as critical regulators of cell survival and death during oxidative stress. ROS can destabilize lysosomes, leading to the release of cathepsins into the cytosol, where they function as proapoptotic mediators and amplify downstream death signalling [69]. Lysosomal destabilization has been implicated in acetaminophen‐ and alcohol‐mediated hepatotoxicity [29, 58, 70]. In the present study, lysosomal integrity was compromised at early time points following IR exposure, as evidenced by reduced LTR fluorescence and increased cathepsin D activity. The absence of significant changes in cathepsin B activity suggests selective involvement of lysosomal proteases rather than nonspecific lysosomal rupture. Importantly, inhibition of CYP2E1 significantly attenuated lysosomal destabilization, directly linking lysosomal injury to CYP2E1‐mediated oxidative stress.

Pharmacological modulation further clarified the role of lysosomes in IR‐related cellular injury. Stabilization of lysosomal function with Baf preserved lysosomal integrity and mitigated downstream mitochondrial damage, whereas ChQ exacerbated lysosomal destabilization and mitochondrial impairment, consistent with previous reports [46–48]. In contrast, mitochondrial stabilization did not prevent lysosomal injury, supporting the interpretation that lysosomal dysfunction represents an early and initiating event during IR exposure.

Mitochondria are central regulators of hepatocyte metabolism, redox balance, and apoptotic signalling [50, 51]. Oxidative stress and lysosome derived proteases can compromise mitochondrial integrity, leading to loss of MMP and activation of apoptotic pathways [52]. In this study, IR exposure resulted in progressive mitochondrial depolarization, which was significantly attenuated by CYP2E1 inhibition and lysosomal stabilization. These findings indicate that mitochondrial dysfunction occurs downstream of CYP2E1 driven oxidative stress and lysosomal injury, rather than as a primary initiating event.

The convergence of lysosomal and mitochondrial alterations ultimately manifested as apoptotic cell death, as evidenced by increased Annexin V positivity, caspase 3 activation, and partial protection afforded by mitochondrial stabilizing agents.

This study has several limitations. It was conducted in an in vitro CYP2E1 overexpressing HepG2 (HepG2‐E47) cell model and confirmed CYP2E1 specificity was tested with only one inhibitor (CMZ); a second CYP2E1 inhibitor or CYP2E1 knockdown would be needed for definitive proof. This study allows mechanistic insights but does not fully recapitulate in vivo hepatic physiology. Although clinically relevant concentrations of INH and RIF were used, in vitro conditions cannot account for systemic pharmacokinetics or chronic drug exposure during tuberculosis therapy. Pharmacological inhibitors were employed to dissect lysosomal and mitochondrial pathways; although well established, potential off‐target effects cannot be excluded, and genetic validation was not performed. Formal normality testing was not performed in this study and parametric tests were used based on standard practice for this class of experiment, and this represents a limitation.

Despite these limitations, the HepG2‐E47 cell line provides a reliable and reproducible system to study CYP2E1‐dependent toxicity, as it retains key hepatocyte functions, enabling functional CYP2E1 activity. The present study further corroborates in vivo evidence that CYP2E1 and NOX contribute to oxidative stress and hepatocellular injury in ATD‐related hepatotoxicity.

Taken together, the findings of this study indicate that IR cotreatment in CYP2E1 overexpressing hepatocytes is associated with oxidative stress driven lysosomal damage, subsequent mitochondrial depolarization, and activation of apoptotic pathways. The use of selective pharmacological modulators highlights the coordinated involvement of CYP2E1 activity, lysosomal dysfunction, and mitochondrial impairment in shaping the cellular response to IR exposure.

To the best of our knowledge, this study is the first to establish, using a genetically defined CYP2E1 overexpressing hepatocyte model combined with organelle specific probes, that lysosomal destabilization is an early, upstream event relative to mitochondrial depolarization in ATD‐related hepatocellular injury, rather than a parallel or downstream consequence. This positions the lysosomal compartment, and specifically cathepsin D release, as a candidate early therapeutic target for preventing progression to mitochondrial‐dependent apoptosis in patients receiving IR therapy, a hypothesis that now warrants testing in primary hepatocytes or in vivo models.

**Nomenclature**
AFU: arbitrary fluorescence units
ATDs: antitubercular drugs
BafA: bafilomycin A1
ChQ: chloroquine
CMZ: chlormethiazole
CsA: cyclosporin A
CYP: cytochrome P450
CYP2E1: cytochrome P450 2E1
DCFH‐DA: 2 ^′^,7 ^′^‐dichlorodihydrofluorescein diacetate
DIA: diazoxide
DILI: drug‐induced liver injury
FBS: fetal bovine serum
GSH: glutathione
H_2_O_2_
: hydrogen peroxide
INH: isoniazid
LTR: LysoTracker Red
MDA: malondialdehyde
MEM: minimum essential medium
MMP: mitochondrial membrane potential
NOX: nicotinamide adenine dinucleotide phosphate oxidase
PI: propidium iodide
PNP: p‐nitrophenol
Rh123: Rhodamine 123
RIF: rifampicin
ROS: reactive oxygen species
SOD: superoxide dismutase
TBARS: thiobarbituric acid reactive substances

## Author Contributions

The co‐first authors D.B. and S.S. conducted research experiments, generated data, curated drafting, and analyzed data. P.S.M., G.K.D., A.C., and A.S. conceptualized, designed the research study, and finalized the draft. D.B. and S.S. made equal contributions and are co‐first authors.

## Funding

This study was supported by the Indian Council of Medical Research (10.13039/501100001411; 5/4/3‐2/2010‐NCD‐II).

## Conflicts of Interest

The authors declare no conflicts of interest.

## Supporting Information

Additional supporting information can be found online in the Supporting Information section.

## Supporting information


**Supporting Information 1** The flow cytometry gating strategy, data acquisition, and analysis are described in the method of Supporting Information Data section.

**Supporting Information 2** Raw, machine‐generated flow cytometry images corresponding to the figures in the main manuscript are provided as Figures S1–S11. Figure S1: Representative flow cytometric gating strategy used for analysis of HepG2‐E47 cells. Figure S2: Representative flow cytometric histograms showing intracellular ROS generation in HepG2‐E47 cells following INH‐RIF treatment. Figure S3: Representative flow cytometric histograms showing modulation of INH‐RIF–induced ROS formation by CMZ in HepG2‐E47 cells. Figure S4: Representative flow cytometric histograms showing lysosomal alterations in HepG2‐E47 cells following INH‐RIF treatment. Figure S5: Representative flow cytometric histograms showing the effect of CMZ on INH‐RIF–induced lysosomal dysfunction in HepG2‐E47 cells. Figure S6: Time‐dependent loss of mitochondrial membrane potential in INH‐RIF–treated HepG2‐E47 cells analyzed by flow cytometry. Figure S7: Effect of CMZ on INH‐RIF–induced mitochondrial membrane potential loss in HepG2‐E47 cells. Figure S8: Effect of different modulators on INH‐RIF–induced mitochondrial membrane potential loss in HepG2‐E47 cells. Figure S9: Flow cytometric evaluation of cell death following INH‐RIF treatment in HepG2‐E47 cells. Figure S10: Effect of CMZ on INH‐RIF–induced cell death in HepG2‐E47 cells. Figure S11: Modulation of INH‐RIF–induced cell death in HepG2‐E47 cells by different pharmacological agents. Detailed legends for all Supporting Information figures are included in the Supporting Information Data file.

## Data Availability

The data used to support the findings of this study are included within the article.
