# Supplementary material for: Unveiling the Cellular Mystery of Isoniazid–Rifampicin (IR) in CYP2E1 Overexpressing HepG2 Cells
Source: Int J Hepatol. 2026 Nov 3;2026:9275461. doi: 10.1155/ijh/9275461 (PMC13732098; doi:10.1155/ijh/9275461)
Supplement: Supplementary file 1 [file IJH-2026-9275461-s001.docx]

**Figure S1**

**Figure S1. Representative flow cytometric gating strategy used for analysis of HepG2-E47 cells.**
(A and C) Initial gating of cell population (P1) based on forward scatter (FSC-A) and side scatter (SSC-A) parameters to exclude debris and dead cells from control and INH+RIF treated samples, respectively.
(B and D) Selection of single cell population (P2) from the gated P1 population using FSC-A versus FSC-H plots to eliminate doublets and cell aggregates in control and INH+RIF treated samples, respectively. The tables below each panel indicate the number of events and percentage of gated populations acquired during flow cytometric analysis.

**Figure S2**

**Figure S2. Representative flow cytometric histograms showing intracellular ROS generation in HepG2-E47 cells following INH-RIF treatment.**

Representative histograms of DCF fluorescence (FL1-H) obtained from control and INH-RIF (IR) treated HepG2-E47 cells at different time intervals (1 h, 3 h, 6 h, and 24 h). M1 and M2 populations were identified based on fluorescence intensity, where M2 represented cells with increased intracellular ROS accumulation. A time dependent increase in the M2 population was observed following IR treatment compared with corresponding controls. Histograms represent original flow cytometer generated outputs using DCF-DA staining. The tables displayed beside each histogram represent the raw quantitative data generated directly from the flow cytometer analysis, including the percentage of gated cells and total cell populations corresponding to M1 and M2 regions.

**Figure S3**

**Figure S3. Representative flow cytometric histograms showing modulation of INH-RIF induced ROS formation by CMZ in HepG2-E47 cells.**

DCF fluorescence (FL1-H) histograms obtained from control, INH-RIF (IR), and IR + CMZ treated HepG2-E47 cells are shown. Cell populations were divided into M1 and M2 regions according to fluorescence intensity, where the M2 population indicated higher intracellular ROS levels. IR exposure increased DCF fluorescence with expansion of the M2 population, while CMZ pretreatment attenuated IR induced ROS accumulation. The tables displayed beside each histogram represent the raw quantitative data generated directly from the flow cytometer analysis, including the percentage of gated cells and total cell populations corresponding to M1 and M2 regions. The overlay panel compares fluorescence distribution patterns among the different experimental groups. Histograms represent original machine generated flow cytometric outputs obtained using DCF-DA staining.

**Figure S4**

**Figure S4. Representative flow cytometric histograms showing lysosomal alterations in HepG2-E47 cells following INH-RIF treatment.**

Representative histograms of LysoTracker Red fluorescence (FL3-H) obtained from control and INH-RIF (IR) treated HepG2-E47 cells at different time intervals. M1 and M2 populations were identified based on fluorescence intensity, where reduction of the M2 population indicated lysosomal dysfunction. A time dependent shift from the M2 population toward the M1 population was observed following IR treatment compared with corresponding controls. Histograms represent original flow cytometer generated outputs obtained using LysoTracker Red staining. The tables adjacent to each histogram show the original quantitative data obtained from flow cytometric analysis, including the percentages of gated and total cell populations within the M1 and M2 regions.

**Figure S5**

**Figure S5. Representative flow cytometric histograms showing the effect of CMZ on INH-RIF induced lysosomal dysfunction in HepG2-E47 cells.**

Representative histograms of LysoTracker Red fluorescence (FL3-H) obtained from control, INH-RIF (IR), and IR + CMZ treated HepG2-E47 cells. M1 and M2 populations were identified based on fluorescence intensity, where reduction of the M2 population indicated lysosomal dysfunction. IR treatment caused a shift from the M2 population toward the M1 population, whereas CMZ pretreatment restored the M2 population indicating attenuation of IR induced lysosomal damage. Histograms represent original flow cytometer generated outputs obtained using LysoTracker Red staining. The tables adjacent to each histogram show the original quantitative data obtained from flow cytometric analysis, including the percentages of gated and total cell populations within the M1 and M2 regions.

**Figure S6**

**Figure S6. Time dependent loss of mitochondrial membrane potential in INH-RIF treated HepG2-E47 cells analyzed by flow cytometry.**

Flow cytometric histograms of Rhodamine 123 fluorescence (FL1-H) obtained from control and INH-RIF (IR) treated HepG2-E47 cells at different time points are shown. Cell populations were divided into M1 and M2 regions according to fluorescence intensity, where the M2 population represented cells with intact mitochondrial membrane potential. IR treatment induced a progressive decrease in the M2 population with a corresponding increase in the M1 population, indicating mitochondrial depolarization. Histograms represent original machine generated outputs obtained using Rhodamine 123 staining. The tables presented alongside each histogram contain the raw flow cytometry data, indicating the proportions of gated and total cell populations distributed within the M1 and M2 regions.

**Figure S7**

**Figure S7. Effect of CMZ on INH-RIF induced mitochondrial membrane potential loss in HepG2-E47 cells.**

Representative flow cytometric histograms of Rhodamine 123 fluorescence (FL1-H) obtained from control, INH-RIF (IR), and IR + CMZ treated HepG2-E47 cells are shown. M1 and M2 populations were identified according to fluorescence intensity, where the M2 population represented cells with intact mitochondrial membrane potential. IR treatment caused a shift from the M2 population toward the M1 population indicating mitochondrial depolarization, whereas CMZ pretreatment restored the M2 population suggesting attenuation of IR induced mitochondrial dysfunction. Histograms represent original machine generated outputs obtained using Rhodamine 123 staining. The tables presented alongside each histogram contain the raw flow cytometry data, indicating the proportions of gated and total cell populations distributed within the M1 and M2 regions.

**Figure S8**

**Figure S8. Effect of different modulators on INH-RIF induced mitochondrial membrane potential loss in HepG2-E47 cells.**

Representative flow cytometric histograms of Rhodamine 123 fluorescence (FL1-H) obtained from control, INH-RIF (IR), IR + CHQ, IR + BAF, and IR + CSA treated HepG2-E47 cells are shown. M1 and M2 populations were identified according to fluorescence intensity, where the M2 population represented cells with intact mitochondrial membrane potential. IR treatment induced a shift from the M2 population toward the M1 population indicating mitochondrial depolarization. CHQ pretreatment further enhanced the M1 population, whereas BAF and CSA treatment restored the M2 population compared with IR treated cells alone. Histograms represent original machine generated outputs obtained using Rhodamine 123 staining. The tables presented alongside each histogram contain the raw flow cytometry data, indicating the proportions of gated and total cell populations distributed within the M1 and M2 regions.

**Figure S9**

**Figure S9. Flow cytometric evaluation of cell death following INH-RIF treatment in HepG2-E47 cells.**

Annexin V-FITC/PI stained dot plots obtained from control and INH-RIF (IR) treated HepG2-E47 cells at different time points are shown. Distribution of cells in different quadrants indicated viable, apoptotic, and necrotic populations. Exposure to IR progressively increased the percentage of Annexin V positive cells over time compared with untreated controls, demonstrating induction of apoptotic cell death. The dot plots and associated data represent original outputs acquired from the flow cytometer. The numerical tables shown next to each histogram represent the original flow cytometric output data, including the percentages of cells present in the M1 and M2 gated populations relative to the total analyzed cells.

**Figure S10**

**Figure S10. Effect of CMZ on INH-RIF induced cell death in HepG2-E47 cells.**

Representative Annexin V-FITC/PI flow cytometric dot plots obtained from control, INH-RIF (IR), and IR + CMZ treated HepG2-E47 cells are shown. IR treatment increased the apoptotic cell population as indicated by enhanced Annexin V positivity, whereas CMZ pretreatment reduced IR induced cell death and increased the viable cell population compared with IR treated cells alone. The dot plots and corresponding data represent original machine generated outputs obtained from flow cytometric analysis. The numerical tables shown next to each histogram represent the original flow cytometric output data, including the percentages of cells present in the M1 and M2 gated populations relative to the total analyzed cells.

**Figure S11**

**Figure S11. Modulation of INH-RIF induced cell death in HepG2-E47 cells by different pharmacological agents.**

Representative Annexin V-FITC/PI flow cytometric dot plots obtained from control, INH-RIF (IR), IR + CMZ, IR + DIA, IR + BAF, and IR + CSA treated HepG2-E47 cells are shown. IR exposure markedly increased the apoptotic cell population, whereas treatment with CMZ, DIA, BAF, and CSA reduced Annexin V positive cells and improved cell viability to varying extents. The dot plots and corresponding quantitative data represent original outputs acquired from flow cytometric analysis. The numerical tables shown next to each histogram represent the original flow cytometric output data, including the percentages of cells present in the M1 and M2 gated populations relative to the total analyzed cells.
