# Supplementary material for: Unveiling the Cellular Mystery of Isoniazid–Rifampicin (IR) in CYP2E1 Overexpressing HepG2 Cells
Source: Int J Hepatol. 2026 Nov 3;2026:9275461. doi: 10.1155/ijh/9275461 (PMC13732098; doi:10.1155/ijh/9275461)
Supplement: Supplementary file 2 [file IJH-2026-9275461-s002.docx]

**Supplementary Data**

**Title: Unveiling the cellular mystery of Isoniazid-Rifampicin (IR) in CYP2E1 overexpressing HepG2 cells**

**Method**:

**Gating Strategy, Data acquisition and analysis in Flow cytometry**:

The flow cytometry is a unique technique for study cell population on the basis of their physical and biochemical properties. For generating accurate and reliable data using this technique, gating of target cell population is essential. In the gating process step by step, we selected the target cell population (P1) by separating dead cells and debris due to their low forward scatter (FSC) and side scatter (SSC) signal (**Figure S1**). Subsequently single cell population (P2) was selected by removing cell aggregates and doublets as depicted in **Figure S1**. Finally, the single cell population was analyzed using fluorescence markers to study intracellular ROS, LMP, MMP and cell death in this study. The raw machine generated data of different experiments are provided

**Result**:

**INH-RIF co-treatment and reactive oxygen species (ROS) formation in HepG2-E47 cells**:

In figure 1 in the main text, 1C depicted intracellular ROS using Flow cytometry. The original raw output images corresponding to Figure 1C, along with the data obtained from flow cytometric analysis of intracellular ROS using DCF-DA in both control and IR treated cells, are presented in **Figure S2**. Representative histograms show fluorescence intensity (FL1) at different time points (1 h, 3 h, 6 h, and 24 h).

In these histograms, there were two population of cells designated as M1 and M2 populations based on their fluorescence intensity. The M1 population exhibited lower DCF fluorescence intensity, whereas the M2 population represented cells with higher DCF fluorescence intensity, indicating increased ROS accumulation. This approach enabled comparison of ROS levels between control and IR-treated cells across different time points.

Figure 2 in the main text demonstrated attenuation of INH-RIF induced oxidative stress in HepG2-E47 cells using CYP2E1 inhibitor. Among these figures, 2B demonstrated overlapping DCF fluorescence profiles of control, IR treated and IR +CMZ treated cells. The original raw output images corresponding to Figure 2B, along with the flow cytometric data of intracellular ROS measured by DCF fluorescence, are presented in **Figure S3**. Histograms show FL1 fluorescence intensity distributions with M1 and M2 populations defined according to fluorescence intensity. IR exposure increased DCF fluorescence, indicating elevated ROS generation, whereas CMZ treatment reduced IR induced ROS levels. The bottom panel shows an overlay histogram comparing fluorescence distributions among control, IR treated, and IR + CMZ treated cells using DCF-DA.

**INH-RIF co-treatment and lysosomal dysfunction in HepG2-E47 cells**:

Figure 3 in the main text, documented INH-RIF induced early lysosomal dysfunction in HepG2-E47 cells through a CYP2E1 dependent mechanism. Among these figures, 3A documented flow cytometric histograms of LTR fluorescence in predetermined time periods. **Figure S4** represented original flow cytometer generated histograms of LysoTracker Red fluorescence along with the data obtained from the instrument. There was a time dependent shift from the M2 population toward the M1 population IR treatment. These histograms represent the direct outputs obtained from the flow cytometer.

Figure 3B in the main text represented flow cytometry histograms analyzing lysosomal dysfunction using LysoTracker Red fluorescence of control, IR, and IR + CMZ treated cells following 90 minutes of IR treatment. **Figure S5** represented the original machine generated histograms along with the corresponding flow cytometric data. IR exposure increased the proportion of cells in the M1 population, indicating lysosomal damage, while CMZ treatment partially restored lysosomal integrity, as reflected by an increase in the M2 population.

**INH-RIF co-treatment and mitochondrial membrane potential (MMP) loss in HepG2-E47 cells**:

In the main text, figure 4 demonstrated the representative figures of CYP2E1 dependent mitochondrial membrane potential (MMP) loss in HepG2-E47 cells due to INH-RIF co-treatment. Among the figures, Figure 4A documented time related loss of MMP in INH-RIF treated cells as evidenced by loss of Rhodamine 123 fluorescence. The original flow cytometer generated histogram along with generated data is presented in **Figure S6**. Furthermore, **Figure S7** presents the original histograms and corresponding flow cytometric data obtained following inhibition of CYP2E1 activity by pretreating the cells with the CYP2E1 inhibitor CMZ prior to INH-RIF treatment.

**Figure S8** shows the original flow cytometer generated histograms along with the corresponding flow cytometric data obtained following pretreatment of HepG2-E47 cells with chloroquine (CHQ), bafilomycin A1 (BAF), and cyclosporin A (CSA) prior to INH-RIF exposure. Histograms of Rhodamine 123 fluorescence demonstrated alterations in MMP following different modulator treatments. IR treatment caused a shift of cell population from M2 toward M1 indicating loss of MMP, whereas pretreatment with BAF and CSA markedly restored the M2 population. Pretreatment with CHQ further increased the M1 population indicating enhanced loss of MMP.

**INH-RIF co-treatment and cell death in HepG2-E47 cells:**

CYP2E1 dependent INH-RIF induced cell death mediated through mitochondrial dysfunction was described in Figure 5 in the main text. Among these figures, representative dot plots of cell death analysis using Annexin V/PI staining were shown. **Figure S9** presents the original dot plots and corresponding data generated from flow cytometer. Similarly, the original dot plots along with data obtained from flow cytometer of figure 5D in the main test are depicted as **Figure S10**.

**Figure S11** depicts the original Annexin V-FITC/PI flow cytometric dot plots together with the corresponding quantitative analysis obtained after pretreating HepG2-E47 cells with CMZ, DIA, BAF, and CSA prior to INH-RIF exposure. IR treatment markedly increased the apoptotic cell population as observed by enhanced Annexin V positivity, while pretreatment with the different modulators attenuated IR induced cell death to varying degrees. The dot plots and associated data represent direct outputs generated from flow cytometric analysis.
